# Efficacy and Safety of Dual Antiplatelet Therapy in Acute Ischemic Stroke With Recurrent Symptoms Post-thrombolysis: A Retrospective Cohort Study From the TriNetX US Collaborative Network

**DOI:** 10.7759/cureus.109056

**Published:** 2026-05-17

**Authors:** Chukwudi Nwogu, James Stupin, Dean Neutel, Steven M Thomas, Lara Tablieh, Yingxing Wu, Nicholas Yelverton, Thomas V Kodankandath

**Affiliations:** 1 Neurology, Carilion Clinic, Roanoke, USA; 2 Neurology, Virginia Tech Carilion School of Medicine, Roanoke, USA; 3 Health Analytics and Research, Carilion Clinic, Roanoke, USA

**Keywords:** acute ischemic stroke, aspirin monotherapy, dual antiplatelet therapy, recurrent neurological symptoms, thrombolysis

## Abstract

Introduction

Recurrent neurological symptoms following thrombolysis in acute ischemic stroke (AIS) pose treatment challenges due to limited evidence guiding antiplatelet therapy. Current practices vary across institutions, reflecting a lack of standardized recommendations. This study uses the TriNetX US Collaborative Network to compare the safety and efficacy of dual antiplatelet therapy (DAPT) versus aspirin monotherapy (AM) in this patient population.

Materials and methods

This retrospective study analyzed de-identified electronic health records from the TriNetX US Collaborative Network, comprising over 66 hospitals. Adult patients aged ≥18 years with AIS who underwent thrombolysis within 4.5 hours of symptom onset between January 2015 and December 2023 were identified. Patients who developed recurrent neurological symptoms between one and 30 days post-thrombolysis and were subsequently treated with either aspirin alone or DAPT (aspirin plus clopidogrel) were included. The following two treatment groups were compared: AM and DAPT. Propensity score matching was performed to balance baseline characteristics between groups. The primary efficacy endpoint was recurrent ischemic stroke between 30 and 90 days, and primary safety endpoints were symptomatic intracranial hemorrhage (SICH) and 90-day all-cause mortality. Outcomes were evaluated using odds ratios and adjusted odds ratios, with statistical significance set at two-sided p<0.05.

Results

In this study, 2,502 patients met the inclusion criteria. After propensity-score matching, 889 patients were included in each treatment group. The incidence of recurrent ischemic stroke was similar between both groups (12.2% in the AM group and 12.6% in the DAPT group). The SICH rate was identical in both cohorts (1.1%). The DAPT cohort had a minor reduction in 90-day mortality compared to the AM cohort (1.6% vs 2.5%), although it was non-significant.

Conclusion

In AIS patients who develop recurrent symptoms after thrombolysis, DAPT did not demonstrate a significant advantage compared to AM in reducing recurrent stroke, SICH, or 90-day mortality. These findings support no observed benefit of routine DAPT escalation in this clinical context and support the need for prospective studies to guide management.

## Introduction

Almost 700,000 ischemic strokes occur annually in the United States, with a quarter of these affecting individuals who have previously experienced a stroke [1]. Following an acute ischemic stroke (AIS), intravenous thrombolysis using tissue plasminogen activator (tPA) must be administered within 4.5 hours of symptom onset to enhance functional outcomes [1]. However, even after the use of tPA, re-occlusion may occur, resulting in recurrent neurological deterioration in nearly 14% of patients [2-4]. Treatment approaches for these cases vary between facilities due to the absence of guideline-directed therapies.

While the International Stroke Trial demonstrated the effectiveness of aspirin monotherapy (AM) for post-stroke care, re-occlusion introduces additional treatment-associated risk for the patient through interventional and thrombolytic routes [5]. The Stenting and Aggressive Medical Management for Preventing Recurrent Stroke in Intracranial Stenosis (SAMMPRIS) trial followed, establishing aggressive medical therapy to be the primary mechanism of treatment in patients with intracranial stenosis [6]. This made antiplatelet therapy the standard of treatment, but led to ongoing debate about the proper use of dual antiplatelet therapy (DAPT), resulting in inconsistent application across institutions [7,8].

Multiple meta-analyses and large randomized trials have consistently shown that short-term DAPT, typically administered for 21-90 days following minor ischemic stroke or high-risk transient ischemic attack (TIA), increases the risk of major bleeding, including symptomatic intracranial hemorrhage (SICH), compared to AM; however, the absolute risk remains low. For example, pooled analyses report SICH rates with DAPT in the range of 0.2%-0.5%, with relative risk for intracranial hemorrhage (ICH) or major bleeding events generally between 2% and 3%, but with absolute risk increases of less than 1% over short durations [9-12]. The American Stroke Association, in its 2021 guideline, specifically notes that short-term DAPT is associated with a modest increase in SICH risk, but the absolute event rates are low and comparable to those seen in landmark trials [10].

Support for DAPT treatment comes in part from the Clopidogrel and Aspirin for Reduction of Emboli in Symptomatic Carotid Stenosis (CARESS) and Clopidogrel plus aspirin for infarction reduction in acute stroke or transient ischaemic attack patients with large artery stenosis and microembolic signals (CLAIR) trials, which reported reduced microembolic signals among patients taking DAPT for cerebrovascular stenosis [13,14]. Additionally, the Clopidogrel in High-Risk Patients with Acute Nondisabling Cerebrovascular Events (CHANCE) trial demonstrated the benefit of DAPT in reducing symptom recurrence in acute minor ischemic stroke (AMIS) while the Antiplatelet vs R-tPA for Acute Mild Ischemic Stroke (ARAMIS) trial indicated that DAPT was non-inferior to intravenous tPA in cases of minor, non-disabling AIS [15-18]. Other studies have highlighted a reduction in ischemic events but with varying reports of hemorrhagic risk [19,20]. Zhao et al. (2019) further supported the results of the CHANCE trial, but had the same weakness of limited population diversity, thereby restricting the generalizability of findings [17,20]. The Platelet-Oriented Inhibition in New TIA and Minor Ischemic Stroke (POINT) trial attempted to address this shortcoming but was stopped at 84% recruitment due to adverse hemorrhage events [7,21]. 

These prior investigations on DAPT primarily focused on patients with TIA or AMIS, leaving uncertainty regarding its utility for patients experiencing recurrent neurological symptoms after thrombolysis for AIS. Preliminary results have shown that stroke recurrence and hemorrhage risk are reduced in patients who receive greater doses of DAPT compared to tPA, but necessitate follow-up studies to generalize these results [22]. To address this gap, we propose leveraging the TriNetX US Collaborative Network to evaluate the safety and efficacy of DAPT when compared with single antiplatelet therapy in patients experiencing recurrent neurological symptoms after being treated for AIS with tPA.

This article was previously presented as a poster presentation at the Society of Vascular and Interventional Neurology 2025 Annual Meeting on November 22, 2025.

## Materials and methods

Data source

De-identified patient data were obtained from the TriNetX US Collaborative Network, which includes data from over 66 hospitals [23]. TriNetX is a global health research platform that integrates clinical data from more than 140 million patients across more than 100 healthcare organizations. The network complies with the Health Insurance Portability and Accountability Act (HIPAA) and is certified under ISO 27001:2022 for its Information Security Management System. Data for this study were collected on May 24, 2025. This study was exempt from informed consent. All analyses were performed within the secure TriNetX analytics environment.

Patient selection

Patients aged ≥18 years with AIS between January 2015 and December 2023 were identified. The inclusion criteria required thrombolysis within 4.5 hours and recurrent symptoms between one and 30 days post-thrombolysis (Table 1). Propensity score matching was performed on the characteristics presented in Table 2.

**Table 1:** Codes and query conditions used to identify patients who underwent thrombolysis post-acute ischemic stroke and were subsequently treated with aspirin monotherapy or dual-antiplatelet therapy. ICD-10: International Classification of Diseases, Tenth Revision; CPT: Current Procedural Terminology.

ICD-10/CPT/RxNorm Code	Description
Visit Event: EMER OR IMP	Visit: Emergency (at least 18 years old at event) OR Inpatient Encounter (at least 18 years old at event)
I63	Cerebral Infarction
3E03317 OR 37195	Introduction of Other Thrombolytic into Peripheral Vein, Percutaneous Approach OR Thrombolysis, cerebral, by intravenous infusion
Cannot have I62	Other and unspecified nontraumatic intracranial hemorrhage
Any instance that meets the following criteria that occurred within 1 to 30 days of the above (Between Jan 1, 2015 and Dec 31, 2023)	
I63.9 OR I69.3	Cerebral infarction, unspecified OR Sequelae of cerebral infarction
1191 OR 1191 AND 32968	Aspirin OR Aspirin AND Clopidogrel

**Table 2:** Codes used and description of characteristics used for propensity score matching. NIHSS: National Institutes of Health Stroke Scale; ICD-10: International Classification of Diseases, Tenth Revision; CPT: Current Procedural Terminology.

ICD-10/CPT/RxNorm Code	Description
Diagnosis	
I10	Essential (primary) hypertension
I20-I25	Ischemic heart diseases
I50	Heart failure
I48.91	Unspecified atrial fibrillation
E78.5	Hyperlipidemia, unspecified
N18	Chronic kidney disease
K76	Other diseases of liver
E08-E13	Diabetes mellitus
E66	Overweight and obesity
J44	Other chronic obstructive pulmonary disease
G45	Transient cerebral ischemic attacks and related syndromes
R29.70	NIHSS score 0-9
R29.71	NIHSS score 10-19
R29.72	NIHSS score 20-29
E03.9	Hypothyroidism, unspecified
Medications	
1191	Aspirin
32968	Clopidogrel
BL110	Anticoagulants

Treatment groups

Eligible patients were categorized into two treatment groups: AM and DAPT (aspirin and clopidogrel). Treatment regimens included varying dosages and administration protocols for aspirin and clopidogrel. Outcomes were defined as 30- to 90-day recurrence of ischemic stroke, 90-day ICH, and 90-day mortality. Outcome codes are presented in Table 3.

**Table 3:** Codes used to identify recurrent ischemic stroke, intracranial hemorrhage, and 90-day mortality within the TriNetX database. ICD-10: International Classification of Diseases, Tenth Revision; CPT: Current Procedural Terminology.

Outcomes	ICD-10/CPT/RxNorm Code
Recurrence of Ischemic Stroke	I63.50, I63.8, I63.81
Intracranial Hemorrhage	I62 OR I61.9
90-day Mortality	Demographics: Deceased

Comparison of effectiveness and safety

The primary efficacy outcome was the recurrence of any ischemic stroke within 90 days. Primary safety endpoints included 90-day ICH and 90-day mortality. 

Statistical analysis plan

Numeric data was described using measures of central tendency and variation. Differences between comparison groups were assessed using t-tests or non-parametric equivalents for continuous variables, and chi-squared tests or equivalents for categorical variables.

Primary outcomes were evaluated using odds ratios (OR) and adjusted odds ratios (aOR). The OR were calculated with the DAPT group as the reference. Statistical significance was set at p<0.05 for two-sided tests. Given our retrospective observational study design, an a priori power analysis was not conducted; sample size represents the number of eligible patients during the study period. 

## Results

**Figure 1:**
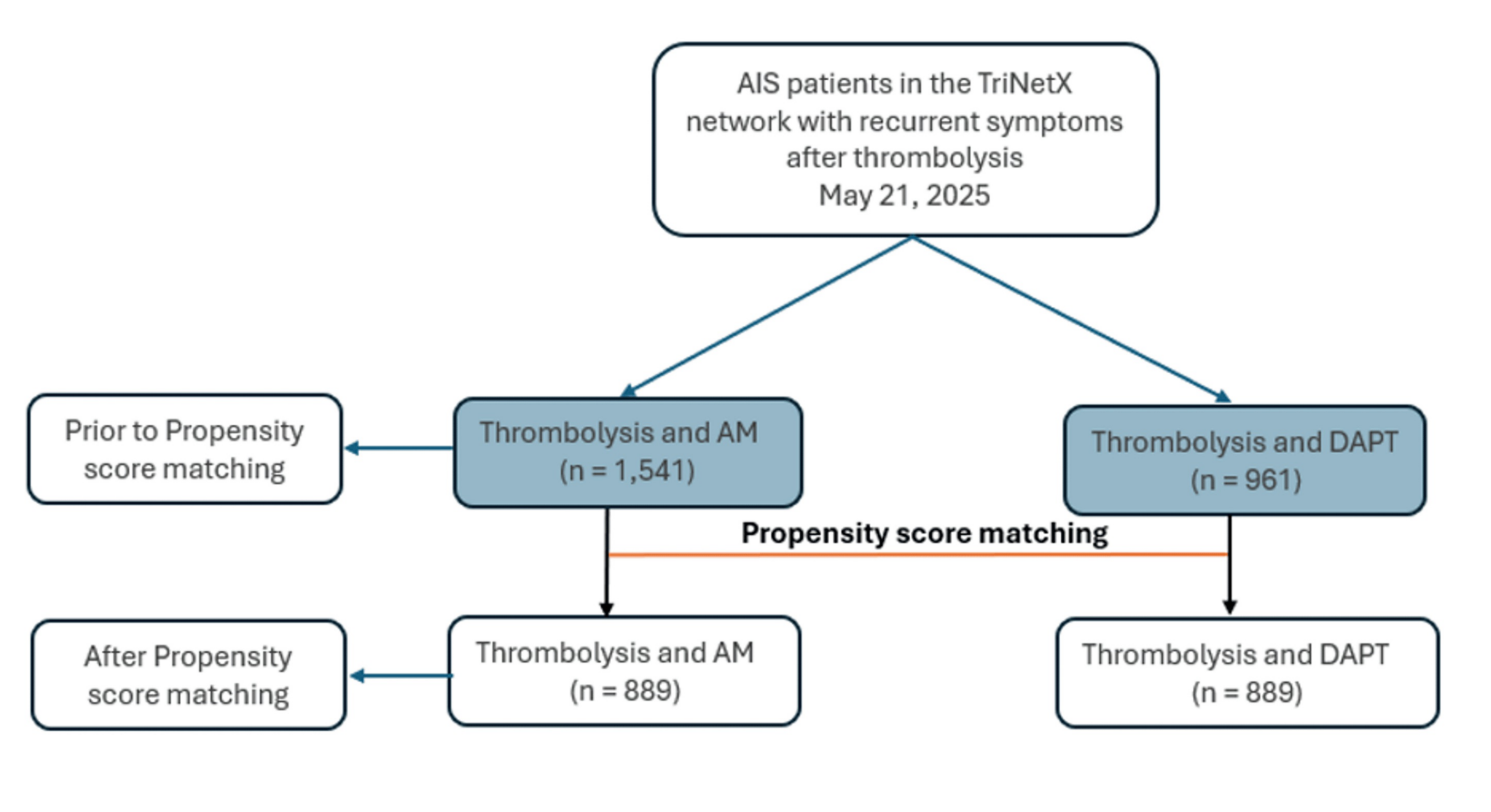
Flow diagram of acute ischemic stroke patients with recurrent symptoms after thrombolysis who received aspirin or aspirin and clopidogrel. AIS: acute ischemic stroke, AM: aspirin monotherapy, DAPT: dual antiplatelet therapy.

On May 21, 2025, 1,541 patients who received aspirin alone and 961 who received aspirin plus clopidogrel after intravenous thrombolysis for AIS were identified between January 1, 2015 and December 31, 2023 (Figure 1).

Before propensity score matching, the AM (n=1,541) and DAPT (n=961) groups differed significantly across several demographic and clinical variables (Table 4). The DAPT group had a higher proportion of men (53.4% vs. 48.9%, p=0.030) and a greater prevalence of comorbid conditions such as essential hypertension (89.0% vs. 83.5%, p<0.001), hyperlipidemia (76.1% vs. 64.0%, p<0.0001), diabetes mellitus (47.1% vs. 40.0%, p<0.001), and ischemic heart disease (39.2% vs. 33.2%, p=0.002). In contrast, atrial fibrillation was more common in the AM group (26.2% vs. 12.2%, p<0.0001).

**Table 4:** Baseline characteristics of patients who underwent intravenous thrombolysis for AIS and were treated with aspirin monotherapy or dual anti-platelet therapy before and after propensity matching. AIS: acute ischemic stroke, AM: aspirin monotherapy, DAPT: dual antiplatelet therapy, NIHSS: National Institutes of Health Stroke Scale, PSM: propensity scoring matching; SD: standard deviation; SMD: standardized mean difference.

Variable % (N)	Initial Population AM (n=1541)	Initial Population DAPT (n=961)	Initial Population * (p-* value)	PSM Population AM (n=889)	PSM Population DAPT (n=889)	PSM Population* * (*p-* value)	PSM Population SMD
Age, years, mean±SD	65.3±13.4	66.3±12.1	0.067	65.4±13.0	65.8±12.2	0.549	0.028
White	58.7 (905)	59.4 (571)	0.733	60.4 (537)	59.1 (525)	0.562	0.028
Male	48.9 (754)	53.4 (513)	0.030	53.4 (475)	52.5 (467)	0.704	0.018
Female	43.5 (671)	37.1 (356)	0.001	38.3 (340)	38.5 (342)	0.922	0.005
Black or African American	20.1 (310)	20.8 (200)	0.675	21.3 (189)	21.0 (187)	0.908	0.006
Unknown Race	10.1 (156)	12.1 (116)	0.128	10.2 (91)	11.6 (103)	0.361	0.043
Unknown Gender	7.5 (116)	9.6 (92)	0.071	8.3 (74)	9.0 (80)	0.613	0.024
Hispanic or Latino	5.1 (78)	6.8 (65)	0.074	6.4 (57)	6.6 (59)	0.848	0.009
Asian	6.6 (102)	4.9 (47)	0.076	5.4 (48)	5.3 (47)	0.916	0.005
Native Hawaiian or Other Pacific Islander	2.8 (43)	1.0 (10)	0.003	1.1 (10)	1.1 (10)	1.000	<0.0001
American Indian or Alaska Native	0.6 (10)	1.0 (10)	0.285	1.1 (10)	1.1 (10)	1.000	<0.0001
Aspirin	87.7 (1351)	95.2 (915)	<0.0001	89.8 (798)	95.1 (845)	<0.0001	0.201
Anticoagulants	76.1 (1177)	77.0 (740)	0.720	77.5 (689)	76.8 (683)	0.74	0.016
Clopidogrel	4.8 (74)	83.5 (802)	<0.0001	5.3 (47)	83.7 (744)	<0.0001	2.567
Essential hypertension	83.5 (1287)	89.0 (855)	<0.001	89.2 (793)	88.3 (785)	0.548	0.028
Hyperlipidemia	64.0 (986)	76.1 (731)	<0.0001	74.7 (664)	74.4 (661)	0.870	0.008
Diabetes mellitus	40.0 (616)	47.1 (453)	<0.001	47.8 (425)	45.8 (407)	0.392	0.041
Ischemic heart diseases	33.2 (511)	39.2 (377)	0.002	37.6 (334)	37.4 (332)	0.922	0.005
Overweight and obesity	27.4 (429)	30.5 (293)	0.155	30.7 (273)	29.8 (265)	0.680	0.020
Heart failure	25.5 (393)	21.5 (207)	0.024	22.6 (201)	21.9 (195)	0.732	0.016
Chronic kidney disease	21.4 (329)	23.0 (221)	0.333	21.8 (194)	21.6 (195)	0.954	0.003
Chronic obstructive pulmonary disease	13.1 (202)	15.5 (149)	0.093	15.2 (135)	14.5 (129)	0.689	0.019
Transient cerebral ischemic attack	11.6 (179)	18.9 (182)	<0.0001	15.2 (135)	16.7 (148)	0.399	0.040
Hypothyroidism	14.6 (225)	12.9 (124)	0.233	12.8 (114)	13.1 (116)	0.888	0.007
Atrial fibrillation	26.2 (403)	12.2 (117)	<0.0001	12.7 (113)	13.1 (116)	0.832	0.010
Liver disease	5.0 (77)	3.9 (37)	0.181	3.9 (35)	4.1 (36)	0.904	0.006
NIHSS score 0-9	37.1 (571)	45.3 (435)	0.000	44.0 (391)	43.4 (386)	0.811	0.011
NIHSS score 10-19	21.6 (333)	17.5 (168)	0.012	18.8 (167)	18.2 (162)	0.760	0.014
NIHSS score 20-29	9.0 (139)	5.9 (57)	0.005	6.2 (55)	6.3 (56)	0.922	0.005

Following 1:1 propensity score matching, 889 patients were classified into each group. After matching, baseline characteristics were well balanced across all variables, with standardized mean differences (SMDs) below 0.1 for all key demographics and comorbidities, indicating appropriate covariate balance (Table 4). No statistically significant differences remained between groups in age, sex, race, comorbid conditions, or National Institutes of Health Stroke Scale (NIHSS) stroke severity categories (p>0.05 for all groups).

**Figure 2:**
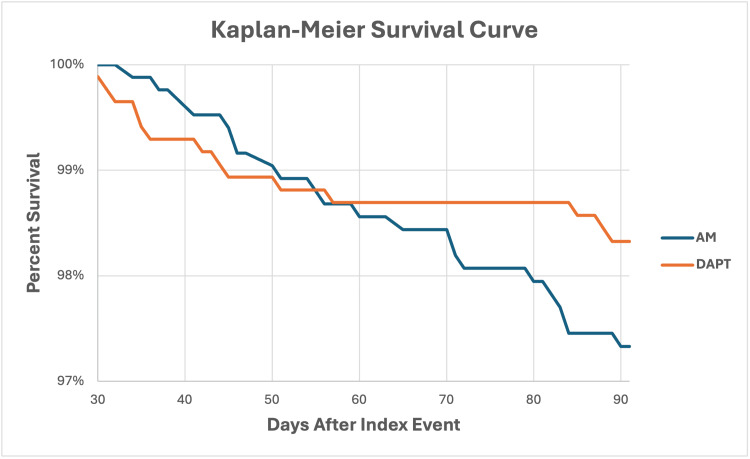
Kaplan-Meier survival analysis of 90-day all-cause mortality in propensity score matched patients treated with aspirin monotherapy versus dual antiplatelet therapy. AM: aspirin monotherapy, DAPT: dual antiplatelet therapy.

The 30- to 90-day recurrence risk of ischemic stroke was 12.2% in the AM group and 12.6% in the DAPT group (odds ratio (OR)=0.969; 95% confidence interval (CI): 0.731-1.285; p=0.8293), with no significant difference between treatment groups (Table 5). The 90-day incidence of ICH was identical in both groups at 1.1% (OR=1.000; 95% CI: 0.414-2.415; p=1.0000), as reported in Table 5. The 90-day all-cause mortality was higher in the AM group (2.5%) than in the DAPT group (1.6%), though this difference was not statistically significant (OR=1.586; 95% CI: 0.806-3.120; p=0.1780), as reported in Figure 2 and Table 5.

**Table 5:** 90-day risk of ischemic stroke recurrence, intracranial hemorrhage, and mortality between AM and DAPT group. AM: aspirin monotherapy, CI: Confidence Interval, DAPT: dual antiplatelet therapy.

Outcomes	% Risk (AM)	% Risk (DAPT)	Odds Ratio (95% CI)
Recurrent Ischemic Stroke	12.2%	12.6%	0.97 (0.76-1.25)
Intracranial Hemorrhage	1.1%	1.1%	1.00 (0.41-2.14)
90-Day Mortality	2.5%	1.6%	1.59 (0.81-3.12)

## Discussion

This retrospective cohort study used the TriNetX US Collaborative Network to compare the efficacy and safety of DAPT (aspirin plus clopidogrel) with AM in patients with AIS who developed recurrent neurological symptoms following thrombolysis. Our findings indicate no statistically significant differences in recurrent ischemic stroke rates, ICH, or 90-day mortality between the two treatment groups. These results suggest that DAPT does not confer a significant advantage over AM in this specific patient population, contrary to the applicability of findings from prior trials focused on TIA or minor ischemic stroke.

Previous studies, such as the CHANCE and POINT trials, demonstrated that short-term DAPT reduces recurrent stroke risk in patients with minor ischemic stroke or high-risk TIA, but at a cost of a modest increase in bleeding risk [18,19]. However, these trials excluded patients who had received thrombolysis and focused primarily on less severe stroke presentations. In a retrospective cohort study by Zhao et al., short-term DAPT (aspirin plus clopidogrel for 21 days) initiated after thrombolysis was associated with a significantly higher rate of favorable 90-day functional outcome, without an observed increase in SICH or 90-day mortality [20]. This suggests that DAPT may improve functional outcomes in selected patients with minor stroke who have received thrombolysis without increasing the risk of major bleeding or death.

It is unclear why patients with recurrent symptoms after thrombolysis did not show a significant benefit in recurrent stroke after DAPT as compared to AM in this study. Patients were propensity-score matched by NIHSS, and the majority fell between 0 and 9 (44.0% and 43.4% for AM and DAPT, respectively, p=0.811), which is similar to earlier studies that reported on mild ischemic stroke [18-20]. However, the addition of patients with higher NIHSS scores up to 29 may have affected the overall result. The risk of ICH was low and similar in the two groups (1.1%), consistent with pooled estimates from prior meta-analyses of short-term DAPT use [9-12]. This finding is reassuring, especially considering the theoretical concern of increased bleeding risk when DAPT is started after thrombolysis [24,25]. Moreover, while DAPT demonstrated a lower 90-day mortality (1.6% vs 2.5%), the effect size was modest and did not reach statistical significance. These findings suggest that any mortality benefit from DAPT in this population, if present, is likely small and requires further investigation.

The ARAMIS trial offers added context, suggesting that DAPT may be non-inferior to alteplase in certain mild, non-disabling stroke cases [15]. While this opens the door to more flexible antiplatelet strategies, its applicability to recurrent post-thrombolysis symptoms remains limited. Additionally, ticagrelor, a more potent P2Y12 inhibitor, has shown some promise in reducing recurrence in AIS or TIA populations [26], and future studies should examine its utility in post-thrombolysis settings with recurrent symptoms.

Given the modest absolute benefit and unchanged hemorrhagic profile observed in our analysis, our findings support a patient-centered approach to antiplatelet selection. Clinicians should carefully weigh individual risk profiles before initiating DAPT.

Limitations

Some limitations of the study are worth noting. Although we applied careful propensity score matching, there is still the possibility of selection bias and residual confounding, particularly due to variations in antiplatelet dosing and administration protocols across hospitals.

The TriNetX platform’s reliance on ICD-10 codes also limited our ability to assess certain clinical details, such as serial NIHSS scores during hospitalization or functional outcomes at 90 days using the modified Rankin Scale. These measures are critical for evaluating clinical progress and functional recovery, potentially omitting early complications or standardized benefits of therapy.

Additionally, reliance on ICD-10 codes precluded direct identification of recurrent strokes as a distinct clinical event, as there is no specific code to distinguish stroke recurrence from previous cerebral ischemia, thereby introducing misclassification bias into the 30- to 90-day recurrence risk of ischemic stroke outcome. 

## Conclusions

In this retrospective study using the TriNetX US Collaborative Network, DAPT did not demonstrate a statistically significant advantage over AM in reducing stroke recurrence, hemorrhagic complications, or mortality in AIS patients with recurrent symptoms after thrombolysis. These findings suggest that DAPT may not provide added benefit in this specific population and reinforce the importance of individualized treatment decisions based on patient comorbidities and bleeding risk. Future prospective, randomized trials are warranted to validate these findings and explore alternative strategies.
